# VCO_2_-derived energy expenditure: do not throw the baby out with the bath water!

**DOI:** 10.1186/s13054-017-1668-3

**Published:** 2017-04-05

**Authors:** Sandra N. Stapel, Paul W. G. Elbers, Heleen M. Oudemans-van Straaten

**Affiliations:** 1 Department of Adult Intensive Care Medicine, Amsterdam, The Netherlands; 2 Research VUmc Intensive Care (REVIVE), Amsterdam, The Netherlands; 3 Institute of Cardiovascular Research (ICaR-VU), Amsterdam, The Netherlands; 4 VU University Medical Center, De Boelelaan 1117, 1181 HV Amsterdam, The Netherlands

With great interest we read the retrospective study performed by Oshima et al. [1] evaluating whether VCO_2_-based energy expenditure (EE-VCO_2_) could be considered as an alternative to EE measured by indirect calorimetry. This study followed several prospective studies reporting good agreement between EE-VCO_2_ and EE measured by indirect calorimetry [2, 3]. Indeed, in their retrospective cohort of 278 mechanically ventilated patients, the authors found a low bias of −48 kcal/day for EE-VCO_2_, when calculated with a fixed respiratory quotient (RQ) of 0.85. They reported 5%-accuracy rates of 46% and 10%-accuracy rates of 77% for EE-VCO_2_. This indicates that EE-VCO_2_ is unreliable in some patients, likely due to extreme RQs or to high ventilator rates as observed in young children [4]. The weak spot of using EE-VCO_2_ is that an RQ has to be assumed in order to derive the unknown oxygen consumption (VO_2_) needed to calculate EE according to the Weir formula: EE(kcal/day) = 1.44 × (3.94 × VO_2_(mL/min) + 1.11 × VCO_2_(mL/min)).

However, despite high 10%-accuracy rates, the authors considered a 10% difference in measured and calculated EE clinically unacceptable and concluded that EE-VCO_2_ should not be considered as an alternative to EE measured by indirect calorimetry.

First and foremost, we agree with the authors that indirect calorimetry remains the gold standard for assessment of EE in mechanically ventilated patients. However, we regret that the authors failed to mention that EE-VCO_2_ should be considered as the best alternative for clinicians not having access to indirect calorimetry. Unfortunately, few units have indirect calorimetry available and, more importantly, the most validated system (Deltatrac) is no longer being manufactured and new devices are not accurate [5]. Many ICU clinicians still rely on predictive equations that have repeatedly proven to be inaccurate, leading to deleterious over- and underfeeding. The results of the Oshima study underscore findings in prospective studies in ICU patients that EE-VCO_2_ has good accuracy and is superior to predictive equations [2, 3]. Furthermore, when using built-in capnographs and flow meters, VCO_2_ is available from the ventilator and EE-VCO_2_ can be used to assess EE continuously. Continuous measurement is important because EE varies over the day and during ICU stay. This is an advantage of EE-VO_2_ over the short-term EE measurements by indirect calorimetry.

Therefore, awaiting new, affordable, and accurate indirect calorimeters, EE-VCO_2_ appears to be the best alternative in spite of its known limitations. Thus, the use of EE-VCO_2_ assuming an RQ of 0.85, rather than applying predictive equations, is currently recommended to reduce over- and underfeeding.

## Abbreviations

EE: Energy expenditure
EE-VCO_2_: Carbon dioxide production based energy expenditure
ICU: Intensive care unit
RQ: respiratory quotient
VCO_2_: Carbon dioxide production
VO_2_: Oxygen consumption
